# Minimizing sedentary behavior (without increasing medium-to-vigorous exercise) associated functional improvement in older women is somewhat dependent on a measurable increase in muscle size

**DOI:** 10.18632/aging.202265

**Published:** 2020-12-03

**Authors:** Dale Grant, David Tomlinson, Kostas Tsintzas, Petra Kolić, Gladys Onambélé-Pearson

**Affiliations:** 1 Research Centre for Musculoskeletal Science and Sports Medicine, Department of Sports and Exercise Sciences, Manchester Metropolitan University, Manchester, UK; 2 MRC Versus Arthritis Centre for Musculoskeletal Ageing Research, School of Life Sciences, Faculty of Medicine and Health Sciences, The University of Nottingham Medical School, Queen's Medical Centre, Nottingham, UK

**Keywords:** DEXA, gait speed, LIPA, sedentary behaviour fragmentation, vastus lateralis

## Abstract

The optimal pattern of sedentarism displacement and mechanisms underlying its health effects are poorly understood. Therefore, the aim of this study was to quantify muscle-tendon adaptation in response to two different sedentarism displacement interventions and relate any adaptations to functional outcomes. Thirty-four older women (73±5yrs) underwent skeletal muscle-tendon size and functional assessments. Participants were randomly allocated to: Sedentary behavior fragmentation (SBF), Light intensity physical activity (LIPA), or Control groups. Measures were taken at weeks 0 and 8. Gait speed significantly increased (p=0.003), in both experimental groups (SBF: 0.06 ± 0.08m/s, 6±10%, LIPA: 0.06 ± 0.07m/s, 6±6%), but not control (-0.02 ± 0.12m/s, -2±9%). Accordingly, the relative change in Vastus Lateralis muscle volume, accounted for 30% (p=0.027), and 45% (p=0.0006) of the explained variance in the relative change in gait speed, for SBF and LIPA respectively. Gastrocnemius Medialis fascicle length changes were positively associated with gait speed changes, following LIPA exclusively (R^2^= 0.50, p=0.009). This is the first study to show SBF and LIPA are adequate loading in older women, with related muscle adaptation and clinically relevant gait speed improvements. Such adaptations appear similar irrespective of whether sedentarism displacement is prescribed in a single bout (LIPA) or in frequent micro-bouts (SBF).

## INTRODUCTION

Sedentary behavior is characterized by low energy expenditure, and a seated/ reclined posture during waking hours [1]. Sedentary time appears hazardous above 8h/day [2, 3] with the achievement of current moderate-to-vigorous physical activity (MVPA) recommendations [4] being insufficient to offset high sedentary time [5]. Accordingly, light intensity physical activity (LIPA) displays a strong inverse correlation with sedentary behavior [6], suggesting LIPA displacement may contribute to the detrimental effects of sedentary time. Furthermore, a prolonged sedentarism accumulation pattern (longer sitting bouts) is associated with worse health outcomes compared to a more fragmented pattern (shorter sitting bouts) [7].

Sedentary time is higher among older adults [8] and is strongly associated with a myriad of poor health outcomes [9–14], most notably compromised physical function [15–18]. The association between sedentary time and compromised function is exacerbated in frail individuals [13], and those accumulating sedentary time in a prolonged pattern [19], which also appears independent of concurrent MVPA time [20]. One mechanism potentially mediating such detriments is muscle tendon complex deterioration. Accordingly, severe disuse induces rapid muscle atrophy [21–23], with sedentary behavior specifically associated with the accelerated age-related loss of muscle mass (pre-sarcopenia) [24]. Furthermore, women tend to exhibit greater anabolic resistance and larger reductions in strength following disuse compared to men [25, 26]. However, the mechano-sensitivity of the human tendon is less clear [27], with only chronic unloading causing tendon atrophy [28]. Nevertheless, short term disuse causes tenocyte mediated detection of force-induced deformations [29] that subsequently trigger catabolic pathways in tendon [30]. Furthermore, alterations in muscle architecture and the force producing capabilities of muscle may also play a role [31–33]. Therefore, sedentary behavior could contribute towards age-related muscle-tendon complex deterioration.

Despite the positive effects of high intensity activity on both muscle [34] and tendon [27, 35, 36] older adults exhibit poor prolonged adherence to MVPA regimens [37–40]. Whilst it may be rational to assume lower intensity activity may not produce a sufficient muscle-tendon adaptation stimulus, evidence for/against this idea is scarce. Indeed, a body of work suggests the necessity for high intensity loading [35, 39], whilst another suggests that older women in particular would benefit from lower intensity loading [41]. Nevertheless, low intensity training has still been shown to stimulate muscle hypertrophy [42], contributing to enhanced strength [43, 44] and physical function [45]. Equally, increases in daily LIPA have been shown to change muscle architecture at rest [46], increase muscle mass [47, 48] and improve physical function [49] in older adults generally, but especially in frail individuals [44, 48–50]. Thus, the potential for LIPA to generate comparable physiological responses relative to more conventional high intensity loading is a somewhat recent theorem, supported by previous observations whereby older adults engaging in low frequency stair climbing exhibit significantly reduced mortality [51]. Therefore, due to the relative surge in physical demands that LIPA seems to generate in older adults closer to the lower limits of their physiological reserve, such activity may reach an appropriate loading threshold required for muscle-tendon complex hypertrophy.

Specifically, displacing sedentary behavior with LIPA improves balance [52] and enhances both gait speed [53] and sit-to stand ability [50, 52] in older adults. Interestingly, acute muscle activity during LIPA appears higher in the *Triceps Surae* compared to the knee extensors [54], which is reasonable given the key role such muscles play in maintaining upright balance [55] and ambulation [56]. Considering gait speed improvements, this ultimately suggests that the *Triceps Surae* may undergo greater adaptation following SB displacement, and thus should be considered a primary target for investigation. Nevertheless, previous interventions have failed to adequately control for the pattern of prescribed LIPA, meaning sedentary behavior fragmentation [repeated interruption of prolonged sitting with frequent sit-to-stand transitions and LIPA breaks (SBF)] may have still caused sufficient knee extensor adaptation. However, muscle-tendon complex hypertrophy following LIPA is likely to be small in magnitude given that tendon has a relatively slow turnover rate [27, 57], and lower activity volumes generally stimulate less muscle hypertrophy [58]. Nevertheless, despite muscle size not being a strong predictor of gait speed in older adults, it remains a significant predictor [59, 60], which may ultimately indicate that minor changes in muscle-tendon complex size can still mediate functional improvement following sedentary behavior displacement with light activity in older adults.

Therefore, the aim of the current study was to quantify muscle-tendon complex hypertrophy in response to two different LIPA interventions in older females and relate any such adaptations to functional outcomes. The first intervention would emulate traditional exercise through a single daily LIPA bout, whereas the second would implement the same amount of LIPA as in the first group but be spread throughout the day (SBF). It was hypothesized that both interventions would induce muscle-tendon complex hypertrophy and improve overall lean body mass, thus translating to improved function (such as gait speed). It was further hypothesized that muscular adaptation would be disproportionately observed in the *Triceps Surae* [(*Gastrocnemius Medialis* (*GM*)/ *Gastrocnemius Lateralis* (*GL*)] group compared to the knee extensor group [*Vastus Lateralis* (*VL)*]. Finally, we hypothesized SBF would induce comparable muscle-tendon complex hypertrophy and functional improvement to those attained through continuous LIPA.

## RESULTS

### Descriptive characteristics of participants at baseline

The 34 older women were matched at baseline for all outcome variables of interest (Table 1). Briefly, there was no statistically significant difference between the three groups at study onset for either *GM*, *GL,* or *VL* regional anatomical cross sectional area (CSA), total muscle volumes, or fascicle length (Lf). Furthermore no baseline differences were observed for *Achilles Tendon* average or regional CSA, nor any of the segmental DEXA-derived body composition outcome variables (Table 2). Only *VL* fascicle pennation angle (FPA) (p≤0.001) and *VL* physiological cross-sectional area (PCSA) (p=0.005) exhibted signficant differences between groups at baseline.

**Table 1:** Baseline characterstics of the study sample.

		**SBF (n=13)**	**LIPA (n=13)**	**Control (n=8)**	**Whole sample (n=34)**
Age (years)		74 ± 5	74 ± 6	70 ± 3	73 ± 5
Height (m)		1.59 ± 0.07	1.61 ± 0.07	1.58 ± 0.1	1.60 ± 0.06
Mass (Kg)		68.8 ± 11.7	65.6 ± 8.9	65.4 ± 9.7	66.8 ± 10.1
Dual X-Ray absorptiometry derived data	Sarcopenic index	6.32 ± 0.81	5.97 ± 0.80	5.92 ± 0.81	6.08 ± 0.80
Sarcopenic Categorization	Proportion classified as Non-sarcopenic (Pre-sarcopenic/Low functional performance)	85% (0%/15%)	77% 23%/0%)	88% 12%/0%)	83% (12%/5%)
Physical Behavior classification	Proportion classified as Sedentary (Non-sedentary)	92% (8%)	100% (0%	75% (25%)	91% (9%)
Physical Behavior	Sedentary Behavior (h/24h)	9.6 ± 1.3	9.5 ± 1.0	8.3 ± 1.8	9.3 ± 1.4
	Light intensity physical activity (h/24h)	2.0 ± 0.8	2.1 ± 0.4	2.2 ± 0.7	2.1 ± 0.5
	Moderate to vigrous physical activity (h/24h)	3.0 ± 1.0	2.5 ± 0.8	3.6 ± 1.1	3.0 ± 1.0

Participant characteristics values are means ± SD.

**Table 2:** Changes in skeletal muscle-tendon size, muscle architecture, lean body mass, and functional performance.

		**SBF (n=13)**			**LIPA (n=13)**			**Control (n=8)**		
		**Pre**	**Post**	**Absolute change (Δ% change)**	**Pre**	**Post**	**Absolute change (Δ% change)**	**Pre**	**Post**	**Absolute change (Δ% change)**
Gastrocnemius Medialis	Total Volume (cm^3^)	195.1 ± 33.3	186.2 ± 41.6	-8.9 ± 27 (-5 ± 13%)×	215.0 ± 49.0	211.2 ± 46.0	-3.8 ± 21.2 (-1 ± 9%)×	175.0 ± 31.6	196.3 ± 30.5	21.3 ± 12.6 (13 ± 8%)×
	FPA (°)	20 ± 3	19 ± 2	-1 ± 4 (-2 ± 22%)	18 ± 3	18 ± 2	0 ± 2 (-1 ± 9%)	18 ± 3	19 ± 3	1 ± 3 (0 ± 16%)
	Fascicle Length (cm)	5.5 ± 0.7	5.2 ± 0.6	-0.2 ± 0.6 (-4 ± 10%)×	5.7 ± 0.7	5.9 ± 0.5	0.3 ± 0.4 (5 ± 8%)×	6.0 ± 0.7	6.0 ± 0.7	-0.1 ± 0.4 (-1 ± 7%)×
	PCSA (cm^2^)	36.0 ± 6.7	35.7 ± 8.2	-0.2 ± 6.3 (0 ± 17%)	37.5 ± 8.3	35.5 ± 8.0	-2.0 ± 4.5 (-5 ± 12%)	29.7 ± 5.8	33.4 ± 4.7	4.0 ± 2.5 (15 ± 12%)
Gastrocnemius Lateralis	Total Volume (cm^3^)	130.8 ± 30.7	134.9 ± 39.1	4.2 ± 25.3 (4 ± 19%)	135.2 ± 29.5	137.5 ± 25.4	2.4 ± 26.2 (4 ± 21%)	133.5 ± 28.6	138.3 ± 21.0	4.8 ± 22.0 (5 ± 13%)
Vatus Lateralis	Cross sectional area at 50% muscle length (cm^2^)	41.3 ± 9.1	41.7 ± 9.7	0.4 ± 5.3 (1 ± 14%)	37.8 ± 6.0	37.8 ± 10.5	-0.1 ± 7.2 (-1 ± 18%)	34.8 ± 8.2	33.4 ± 7.4	-1.4 ± 2.8 (-4 ± 9%)
	Total Volume (cm^3^)	464.2 ±191.55	446.6 ± 201.8	0.5 ± 88.9 (0 ± 18%)	448.1 ± 95.2	435.6 ± 128.8	-17.4± 100.0 (-4± 23%)	395.2 ± 79.21	402.2 ± 78.45	2.7 ± 31.0 (1 ± 7%)
	FPA (°)	**19 ± 3**	18 ± 3*	2 ± 3 (11 ± 21 %)	**16 ± 2**	16 ± 3*	-1 ± 3 (-4 ± 18 %)	**14 ± 3**	14 ± 2*	-3 ± 2 (-18 ± 9 %)
	Fascicle Length (cm)	5.4 ± 1.3	5.6 ± 1.3	-0.1 ± 0.9 (0 ± 16 %)	5.2 ± 1.1	5.4 ± 1.4	0.2 ± 0.5 (5 ± 9%)	6.3 ± 1.2	6.3 ± 1.8	-0.4 ± 1.0 (-8 ± 12%)
	PCSA (cm^2^)	**89.8 ± 21.9**	92.4 ± 24.1	4.2 ± 24.7 (5 ± 30%)	**79.2 ± 16.1**	73.6 ± 22.4	-7.9 ± 20.3 (-11±23 %)	**58.3 ± 16.5**	58.8 ± 30.5	3.8 ± 11.3 (4 ± 22 %)
Achilles Tendon	Average cross sectional area (cm^2^)	0.78 ± 0.19	0.80 ± 0.20	0.03 ± 0.10 (3 ± 13%)	0.74 ± 0.14	0.74 ± 0.17	0.03 ± 0.11 (4 ± 15%)	0.70 ± 0.17	0.67 ± 0.19	0.01 ± 0.07 (1 ± 9%)
	Cross sectional area at 0cm (cm^2^)	0.86±0.34	0.90±0.31	0.03±0.19 (11±40%)	0.77±0.22	0.79±0.18	0.02±0.31 (13±48%)	0.74±0.20	0.69±0.08	-0.05±0.26 (-7±45%)
	Cross sectional area at 1cm (cm^2^)	0.78±0.19	0.81±0.33	0.03±0.17 (4±24%)	0.82±0.19	0.82±0.25	0.02±0.11 (3±19%)	0.74±0.28	0.78±0.23	0.04±0.17 (3±24%)
	Cross sectional area at 2cm (cm^2^)	0.76±0.14	0.78±0.15	0.02±0.12 (4±17%)	0.81±0.16	0.79±0.29	-0.02±0.11 (-2±15%)	0.70±0.36	0.67±0.25	-0.02±0.15 (-1±21%)
	Cross sectional area at 3cm (cm^2^)	0.72±0.22	0.71±0.11	-0.01±0.12 (-1±19%)	0.65±0.18	0.65±0.21	0.01±0.07 (1±9%)	0.59±0.24	0.65±0.25	0.03±0.15 (3±26%)
Dual X-Ray absorptiometry derived data	Arms Lean Tissue (Kg)	1.86 ± 0.33	1.85 ± 0.29	-0.01 ± 0.15 (0 ± 8%)	1.80 ± 0.22	1.76 ± 0.24	-0.03 ± 0.08 (-2 ± 5%)	1.71 ± 0.24	1.76 ± 0.24	0.05 ± 0.07 (3 ± 4%)
	Legs Lean Tissue (Kg)	6.17 ± 1.24	6.06 ± 0.99	-0.11 ± 0.45 (-1 ± 6%)	5.93 ± 0.77	5.85 ± 0.71	-0.07 ± 0.20 (-1 ± 3%)	5.66 ± 0.83	5.66 ± 0.83	0.01 ± 0.19 (0 ± 3%)
	Total Lean Tissue (Kg)	39.14 ± 5.92	38.91 ± 5.59	-0.23 ± 1.46 (-1 ± 3%)	37.10 ± 4.07	36.70 ± 3.88	-0.0 ± 1.13 (-1 ± 3%)	36.75± 4.05	37.44 ± 4.00	0.68 ± 0.79 (2 ± 2%)
Functional Performance Measures	Gait Speed (m/s)	1.12 ± 0.29	1.16 ± 0.37*	0.06 ± 0.08 (6 ± 10%)	1.12 ± 0.13	1.21 ± 0.18*	0.06 ± 0.07 (6 ± 6%)	1.30 ± 0.10	1.29 ± 0.24*	-0.02 ± 0.12 (-2 ± 9%)
	Eyes open balance (s)	19 ± 11	20 ± 12	1 ± 6 (11 ± 46%)	27 ± 5	27 ± 5	0 ± 4 (6 ± 30%)	26 ± 5	25 ± 8	-1 ± 4 (-7 ± 20%)
	Eyes closed balance (s)	3 ± 2	3 ± 3	0 ± 3 (29 ± 85%)	4 ± 3	5 ± 5	1 ± 2 (33 ± 63%)	3 ± 2	3 ± 1	0 ± 2 (2 ± 56%)

Participant Characteristics values are means ± SD.

Boldened baseline values represent significant baseline differences. * represents a significant time effect. × represents a significant group×time interaction effect.

### Physical behavior

All groups were significantly matched for sedentary behavior, LIPA, and MVPA at baseline (p≥0.05). There was no group×time interaction for sedentary behavior (p=0.41). However, a trend for an effect over time was observed (p=0.08, ɳ_p_^2^=0.21) driven primarily by a decrease in both experimental groups (SBF: -2±15%, LIPA: -4±14%) in contrast to control (4±30%). Promisingly, 8 participants (24%) positively shifted classification from sedentary to non-sedentary (SBF: n=3, LIPA: n=3, CON: n=2) in response to the intervention, with the other 26 participants (74%) remaining stable in their category over time. Furthermore no significant effects were observed for MVPA (p≥0.05) (Please see table 1).

### Sarcopenia categories

All groups were significantly matched at baseline for categories of sarcopenia status (p=0.18), where 82%, 12%, and 6% were categorized as non-sarcopenic, pre-sarcopenic, and low functional performance respectively (Please see table 1). Only one participant positively shifted sarcopenia classification from low-functional performance to non-sarcopenic in response to the SBF intervention, with all other participants remaining stable in their category over time.

### GM, GL, VL volume and PCSA intervention-induced changes

**Figure 1:**
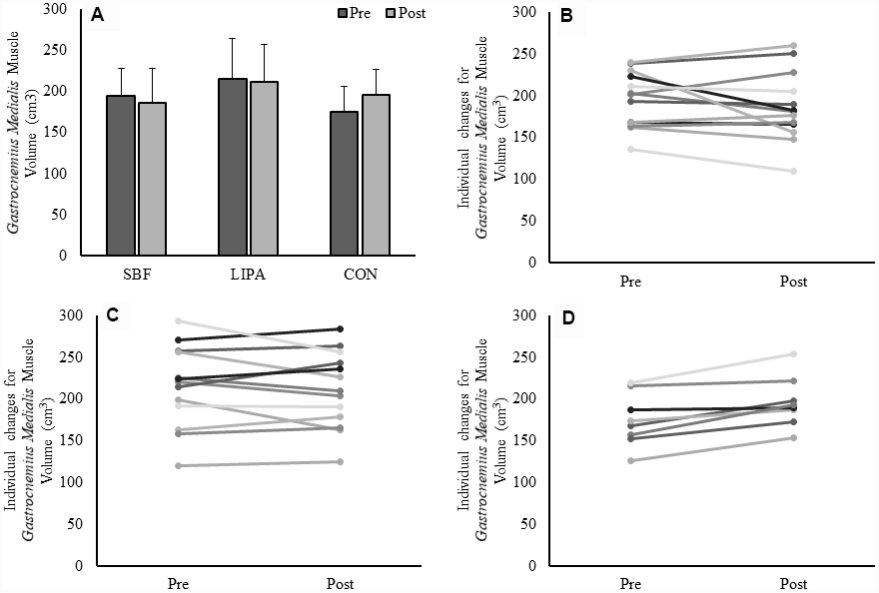
**Changes in *GM* muscle volume from baseline to post-intervention.** Panel (**A**) Group-dependent *GM* muscle volume (Mean ± SD) at pre (week 0) and post intervention (week 8). There was a significant group×time interaction (*p* = 0.014) for GM volume. Panels (**B–D**) represent individual participants changes from baseline to post-intervention, for the SBF, LIPA, and control groups, respectively.

**Figure 2:**
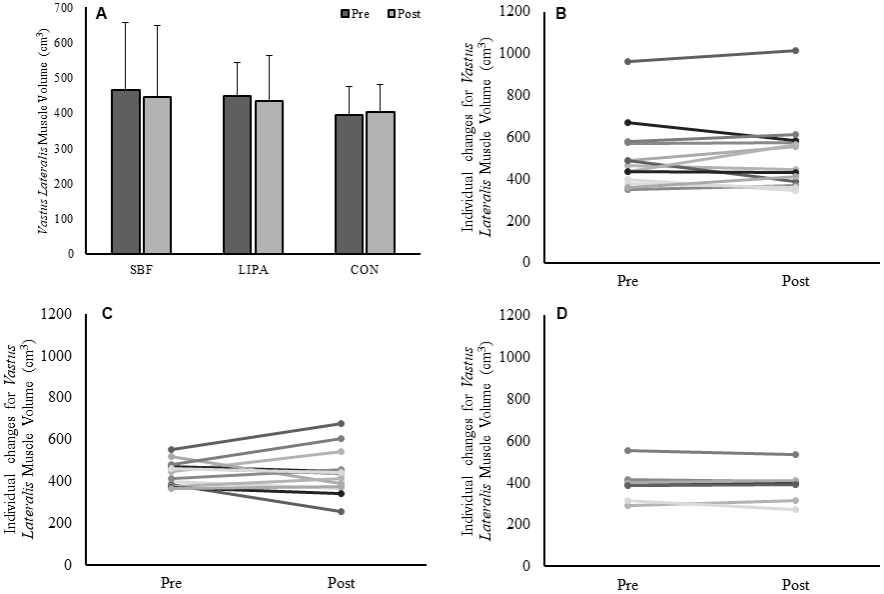
**Changes in *VL* muscle volume from baseline to post-intervention.** Panels (**A**) Group-dependent *VL* muscle volume (Mean ± SD) at pre (week 0) and post intervention (week 8). Panels (**B**–**D**) individual participant changes from baseline to post-intervention for the SBF, LIPA, and control groups respectively.

*GM* volume showed no effect of time (p=0.47), no effect of group (p=0.22) but a group×time interaction (p=0.014, β=0.77, ɳ_p_^2^=0.24), with an increase in the control group *GM* volume being the driver for this interaction (Figure 1A). The associated changes for each group are illustrated in Figure 1B–1D. Similarly, a group×time interaction trend was observed for GM PCSA (p=0.06, β=0.56, ɳ_p_^2^=0.18), with the control group increasing on average (15±12%) in contrast to both SBF (0±17%), and LIPA (-5±12%). However, there were no time, group, or group×time interactions observed for CSA, volume, or PCSA, in the *GL* and *VL* (Please see Table 2 and Figure 2).

### GM and VL resting muscle architecture

*GM* fascicle length (Lf) exhibited a significant group×time interaction effect (p=0.04). The primary driver for this effect was the significant difference between SBF, in which Lf decreased (-0.16±0.55cm, -4±10%), and LIPA, in which it increased (0.35±0.40cm, 5±8%) (p=0.04). Furthermore, once corrected for baseline differences, *VL* FPA exhibited a significant time effect (p=0.010, β=0.75, ɳ_p_^2^=0.20), but not a group×time interaction (p=0.20) (Please see table 2).

### Achilles Tendon dimensions intervention-induced changes

Interestingly for the analysis of the 4 discrete tendon CSA sites, a single trend was observed toward a significant main effect for time at 1cm of *AT* length (p=0.08, ɳ_p_^2^=0.22), but no group×time interaction effect (p=0.99), with all groups increasing to a similar extent (SBF: 4±24%, LIPA: 3±19%, Control: 3±24%). Similarly, average tendon CSA (average of 4 discrete sites) showed no time, group, or group×time interaction (Please see table 2).

### DEXA derived body composition intervention-induced changes

None of the DEXA-derived outcome variables (total lean tissue, arms, legs, and sarcopenic index) exhibited main effects of group, time, nor group×time interactions.

### Functional performance measures intervention-induced changes

**Figure 3:**
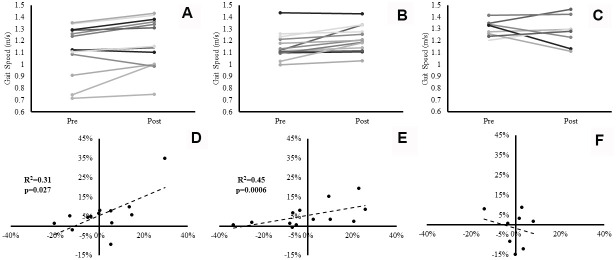
**Individual participants’ changes in gait speed from baseline to post-intervention.** Panels (**A**–**C)** represent individual changes for the SBF, LIPA, and control groups respectively. Panels (**D**–**F**) represent the associations between the relative changes in VL Volume (X axis), and the relative changes in gait speed (Y axis) for the SBF, LIPA, and control groups respectively.

Gait speed exhibited a significant main effect for time (p=0.003, ɳ_p_^2^=0.36). Despite no significant group×time interaction effect (p=0.24), both SBF (0.06 ± 0.08m/s, 6±10%) (Please see Figure 3, Panel A), and LIPA (0.06 ± 0.07m/s, 6±6%) increased from pre to post (Please see Figure 3, Panel B), in contrast to control (-0.02 ± 0.12m/s, -2±9%) (Please see Figure 3, Panel C). However, no significant main effects were observed for postural balance ability.

### Associations between relative changes in muscle-tendon complex size and relative changes in gait speed

There was a significant positive association between % change in *VL* volume and % change in gait speed (p=0.006). Specifically, within the pooled analysis of all participants, the percent change from baseline in *VL* volume significantly (R^2^=0.18, p=0.006), accounted for 18% of the explained variance in relative change from baseline in gait speed. Following sub-analysis by group, the explained variance in gait speed significantly (R^2^=0.31, p=0.027) rose to 31% in the SBF group (Figure 3, Panel D), 45% in the LIPA group (R^2^=0.45, p=0.0006) (Figure 3, Panel E) with no significant variance in the control group (Figure 3, Panel F). Furthermore, there was a significant positive association between the % change in *GM* Lf and % change in gait speed (R^2^=0.24, p=0.004), accounting for 24% of the explained variance. Interestingly, when sub-analyzed by group such an association only persisted for LIPA (R^2^=0.50, p=0.009) and Control (R^2^=0.64, p=0.014), with both groups accounting for similar amounts of the explained variance (LIPA: 50%, CON: 64%). Finally, a significant negative association was observed between the % change in *GM* PCSA and % change in gait speed (R^2^=0.33, p=0.001), accounting for 33% of the explained variance. Following sub-analysis by group, the explained variance in gait speed only persisted for SBF (R^2^=0.46, p=0.010), and LIPA (R^2^=0.37, p=0.014). Both experimental groups accounted for similar amounts of the explained variance (SBF: 46%, LIPA: 37%). No other significant correlations were observed between relative changes from baseline in muscle-tendon complex or DEXA outcomes and relative changes from baseline in functional performance measures.

## DISCUSSION

The aim of the current study was to quantify muscle-tendon complex hypertrophy in response to two LIPA interventions in older women and relate any changes to functional outcomes. Firstly, it was hypothesized that both interventions would induce measurable muscle-tendon complex hypertrophy, improve overall lean body mass, translating into enhanced function. Accordingly, we observed a significant change over time for *VL* FPA, and group-dependent changes over time for *GM* Lf and *GM* muscle volume. Furthermore, gait speed significantly improved in both experimental groups but not control. The % change in gait speed was significantly associated with the % change in *VL* volume (R^2^=18%), and *GM* Lf (R^2^=24%) thereby partially upholding the primary hypotheses. It was further hypothesized that muscular adaptation would be disproportionately observed in the *Triceps Surae* group. We observed localized maladaptation in *GM* volume following both LIPA interventions, with the relative change in *GM* PCSA negatively associated with the percent change in gait speed (R^2^= -33%). Thus, the third hypothesis was partially refuted. Finally, we hypothesized that SBF would induce comparable muscle-tendon complex hypertrophy, and functional improvement, to those of continuous LIPA. *GM* Lf significantly increased in LIPA only, whereas a decrease was observed in SBF. Accordingly, the % change in *GM* Lf was significantly associated with the % change in gait speed for LIPA but not SBF. Nevertheless, gait speed improved by similar magnitudes in both experimental groups, with the relative change in *VL* volume accounting for similar amounts of the explained variance for the relative change in gait speed (SBF:30%, LIPA:45%), thereby upholding the final hypothesis.

Despite the abundance of health benefits that exercise induces [61], older adults exhibit poor long-term adherence to exercise [40]. Furthermore, recommended exercise does not offset the negative effects of high sedentary time [5]. Such limitations create scope for alternative interventions that potentially yield greater long-term efficacy. Accordingly, displacing sedentary behavior with LIPA in older adults, consistently improves physical function [50, 52, 53], however the physiological mechanisms remain undetermined. The current study is the first to examine muscle-tendon complex hypertrophy following sedentary behavior displacement in older females, and link adaptations to functional improvements.

We found that LIPA implementation failed to elicit statistically significant improvements in *GM*, *GL*, or *VL* muscle volume/ PCSA. In contrast, a single bout of low-intensity resistance training (40% 1RM) is sufficient to stimulate myofibrillar protein synthetic response [58]. However, only slow tempo lifting through the entire range of motion, significantly improved quadriceps muscle thickness following 10 weeks of low-intensity resistance training (30-50% 1RM) in older adults [62, 63]. Consequently, prescribed LIPA should have theoretically provided enough intensity, but the lack of direct supervision may have led to variability in movement execution (range of motion/training tempo). Furthermore, low volume (3 sets) low-intensity resistance training appears inferior to high volume (6 sets), regarding the ability to stimulate myofibrillar protein synthetic response in older adults [58], suggesting increasing training volume over time is essential for hypertrophy. LIPA interventions may therefore require to be carried out over longer periods to compensate for the lack of overload. Furthermore, considering the role body weight plays in muscle-tendon complex adaptation [64], variations in participants mass did not allow specific standardization of training load for body weight-based movements. Nevertheless, increasing older adults walking time over 6 months, increases skeletal muscle mass [47, 48]. However, previous LIPA interventions of similar durations similarly did not increase training volume, or manipulate range of motion/training tempo, yet still observed improved function [50]. This suggests improved physical function following sedentary behavior displacement, may occur independent of significant muscle hypertrophy.

Despite observing a group-dependent effect for *GM* volume, this effect was driven through a marked increase in the control group only. Whilst we instructed intervention participants to maintain habitual MVPA, we did prescribe specific instructions to avoid high-speed activities when displacing sedentary behavior, which may have unintentionally reduced habitual gait. Accordingly, plantar flexor muscle activity is affected by alterations in walking speed [65], and increases during faster walking speeds [66]. The control group received no such instruction and thus may have continued receiving the habitual walking stimulus required to elicit GM hypertrophy. Accordingly, MVPA but not LIPA is associated with mid-calf muscle density in older adults [67]. This points to a localized muscular effect following alterations in ambulation (specifically in the *GM*) that was not generalized across the whole leg. Whilst this supports our original hypothesis that muscular adaptation would be disproportionately observed in the *Triceps Surae* group, we failed to anticipate a maladaptation. Nevertheless, we still observed significant gait speed improvements following both interventions, despite an apparently compromised GM volume.

We failed to observe any significant main effects for *Achilles Tendon* size. Extreme low-intensity resistance training (≤20% 1RM), has been shown to enhance strength in older adults [43, 44]. Given the relative surge in intensity such activity likely stimulates in populations close to the lower end of the physiological reserve spectrum, this suggests light activity may reach an appropriate tendon adaptation loading threshold. Accordingly, tenocytes sense loading induced deformations [29], triggering anabolic and catabolic pathways [30]. However, tendon hypertrophy seems dependent upon reaching an intensity threshold (≥40% 1RM) [27, 35, 36]. Therefore, the lack of significant main effects, further questions the likelihood of training-induced alterations in tendon size. However, this does suggest functional adaptation following sedentary behavior displacement, occurs independent of changes in *Achilles Tendon* size.

Displacing sedentary behavior similarly did not alter lean body mass, with no participants shifting pre-sarcopenia categorization post-intervention. In support, moderate term low-intensity resistance training (10-20 weeks, ≤40% 1RM) does not significantly alter lean body mass in older adults [39, 68]. Furthermore, light homebased body weight resistance training failed to induce changes in fat-free mass over 9 months [69]. However, DEXA tends to underestimate the age-related loss of muscle mass compared with MRI [70]. Nevertheless, given minimal alterations in *GM*, *GL*, and *VL* muscle volume, it is unsurprising that lean body mass similarly did not exhibit significant change post intervention. Furthermore, this points to a localized muscular effect of increased ambulation (specifically in the *GM/VL*), that was not generalized across the whole leg. Similar deficiencies (lack of mechanical impulse/overload), that do not appear to be compensated for through longer time frames, likely inhibited lean body mass gains following sedentary behavior displacement.

We did not observe significant changes in postural balance ability. Previous studies examining the association between SB and postural balance in older adults have reported mixed results [18, 71]. Furthermore, only one study exhibited a trend towards improved balance following SB displacement in older adults [52]. However, the latter study utilized a slightly longer intervention period (12 weeks), and only assessed balance through timing single leg stance [52]. The current study also varied the proprioceptive feedback through adding an eyes closed balance assessment [72], although this did not alter the results. Therefore, our findings potentially highlight the insufficiency of SB displacement as an appropriate PA modality for balance improvement. In fact, a minimum of 90 minutes a week of specific balance training is suggested to be the minimum dose response threshold for balance improvement in older adults [73]. Future studies could therefore implement single leg challenges during SB displacement, utilize more nuanced balance assessments (posturography), and employ longer intervention times (≥12 weeks) to further determine if balance/postural sway is impacted through displacing older adults SB time.

Most notably, we did observe significant improvements in gait speed. Gait speed is used as a key diagnostic indicator of low functional performance and severe sarcopenia in older adults [74]. Improvements in gait speed are frequently associated with an increase in daily walking time [49], and time spent performing low-intensity resistance training [42] in older adults. Improved gait speed is also a consistent finding throughout sedentary behavior displacement studies in older adults [50, 52, 53]. Therefore, our results in line with previous research suggest, LIPA can stimulate functional improvement in older adults. Furthermore, given that both experimental groups improved their gait speed to a similar extent, this suggests the act of displacing sedentary behavior time with increased LIPA is the principal factor mediating gait speed improvements, irrespective of the prescribed pattern.

Interestingly, our results do reveal the relative change in gait speed was significantly associated with changes in *VL* volume, accounting for ~18-45% of the observed variance. This further suggests improvements in gait speed may be dependent on ’small’ changes in *VL* muscle size. Accordingly, *VL* muscle size has been identified as a small yet significant independent predictor of fast gait speed in older adults [60]. This is reasonably expected, given that we assessed gait speed through the TUG test, and the knee extensors play a key role in sit-to-stand transitional performance and ambulation in general [75], with previous authors speculating sedentary behavior displacement was specifically improving the ability to mobilize from a seated position [50]. Accordingly, we observed a significant increase in *VL* FPA following SBF (~11%). In support, eight weeks of light dancing similarly increases *VL* FPA in older women (~21%) [46]. Given that increased FPA is associated with increased force transmission [31, 33], this supports positive knee extensor adaptation following SBF. However, only *VL* volume significantly correlated with the change in gait speed, suggesting an exclusive role. Accordingly, muscle volume appears superior to CSA regarding the ability to evaluate age-related differences in muscle strength [76]. Furthermore thigh muscle volume has specifically been associated with muscle power, sit-to-stand ability, and fast gait speed in older adults [59]. The significant negative association between % change in *GM* PCSA and % change in gait speed in both experimental groups also supports this finding. Whereas PCSA represents the amount of sarcomeres in parallel, and thus a muscles maximal force production capabilities [77, 78], gait speed appears more dependent on contraction velocity and the adequate production of muscular power [59].

Our results also revealed that the % change in gait speed was significantly associated with % changes in *GM* Lf. In contrast to FPA and PCSA, Lf accurately represents the amount of sarcomeres in series, and is thus a major determinant of maximum shortening velocity [77]. Specifically, 50% of the difference in maximum shortening velocity between young and old adults is explained by a reduction in *GM* Lf [79]. Our results support this finding given that 50% of the variance for % change in gait speed, was accounted for through changes in *GM* Lf, following the LIPA intervention exclusively. Accordingly, *GM* Lf significantly increased in LIPA (5%), but not SBF (-4%). In support of this finding, eight weeks of light dancing increases *GM* Lf in older women by a similar magnitude (~10%) [46], suggesting a specific mechanism by which continuous LIPA increases gait speed. Walking preferentially stimulates the *Triceps Surae* musculature in older adults [54], suggesting continuous LIPA may have involved greater time spent ambulating in contrast to SBF. Consequently, greater time spent ambulating may have generated the region-specific effect on *GM* Lf. Therefore, together with reduced *GM* PCSA, increased *GM* Lf may represent a shift toward greater contraction velocity capabilities in the *GM*. Ultimately, gait speed improvements following LIPA implementation in older women, appear to be comprehensively mediated through small changes in *VL* muscle volume, as-well as a pattern dependent shift in *GM* Lf. Despite identifying such important mediators, ~76-82% of the variance remains unexplained, suggesting other physiologic mechanisms further mediate gait speed improvements following sedentary behavior displacement. These additional mechanisms likely include alterations in fiber type composition, tendon mechanical properties, as-well as neuromuscular adaptations, which we recommend future studies investigate.

Given that we exclusively recruited older females, this does limit the generalisability of our findings to other populations. However, we see this as a strength given that it was recently shown muscle-tendon complex response to resistance training may be gender dependent [80]. Whilst we acknowledge splitting our small sample into three groups likely reduced our statistical power, we view this as a necessary trade off given the strong study design we employed (accounting for prescribed LIPA pattern, utilizing a control group). Indeed, our achieved sample size (n=34) is in line with previous interventions. Furthermore, given that DEXA underestimates the age-related loss of muscle tissue [70], we view the simultaneous utilization of DEXA and ultrasound muscle assessment as a key strength. Nevertheless, we recommend future interventions utilize longer time frames (>8 weeks), in order to compensate for the limited degree of overload. Our original hypothesis led us to prioritize investigation of the *Triceps Surae* (2 muscles, 3 regional measurement sites per muscle) over the knee extensors (1 muscle, 1 regional measurement site). Thus, being restricted to a single CSA measurement site in the *VL* meant we may have underestimated regional differences in size along the entire length of the muscle [81–83]. Therefore, given the relevance of *VL* volume within our results, we strongly encourage future studies place greater importance on investigating the knee extensors following sedentary behavior displacement, using multiple measurement sites, and further investigation of the *Quadriceps Femoris* as a whole.

In conclusion, displacing sedentary behavior with LIPA (irrespective of prescribed pattern) produces limited muscle-tendon complex adaptations and significant gait speed improvements in older adults. Furthermore, small alterations in *VL* muscle volume explained a large part of the variance in gait speed changes, which were also associated with changes in *GM* fascicle length. Collectively, these findings suggest that LIPA reaches an appropriate loading threshold required to induce clinically impactful functional adaptations. Future studies should investigate other physiologic mechanisms underlying such observed improvements.

## MATERIALS AND METHODS

Thirty-four community dwelling elderly women voluntarily participated in the study (See table 1). Intervention studies manipulating sedentary behavior in older adults are few, and to the authors’ knowledge no published interventions have examined changes in muscle-tendon complex size or lean body mass. Therefore, estimation of required sample size to detect significant changes in the desired outcomes was based upon two points: (a) previous sedentary behavior interventions in older adults that have observed improvements to physical function, have utilized total sample sizes of ~25-38 [50, 52, 53] (b) previous low-intensity resistance training studies in older adults, deemed total sample sizes of 17 [35], and 18 [63], adequate to detect changes in tendon and muscle size respectively. The current achieved sample size of 34 older females, falls within this range. Participants were all recruited from the local community. The study was approved by the local university ethics committee [approval code: 230118-ESS-DG-(2)], and written informed consent obtained prior to any procedures being performed, in line with the declaration of Helsinki. Exclusion criteria included history of lower limb muscle/ tendon/ joint disorders in the past six months, or current suffering from any chronic health condition which could affect the participants ability to independently perform an intervention of increased activity (e.g. cardiovascular disease, uncontrolled diabetes, active cancer, current diagnosis of stroke, Parkinson’s disease, etc). Furthermore, participants who partook in structured progressive resistance training (free weights etc) were also excluded at baseline. Nevertheless, inclusion criteria comprised all habitual physical activity profiles (regardless of meeting recommended MVPA levels). Participants initially visited the laboratories to complete screening/ questionnaires, as-well as undergo familiarization to the gait speed and balance (functional performance) assessments. After seven days, participants returned to the laboratories and underwent overnight rested and fasted, ultrasonographic assessment of the *(GM)*, *Gastrocnemius Lateralis* (*GL*), *Vastus Lateralis (VL)* and *Achilles Tendon (AT)*. Segmental analysis of body composition using DEXA imaging, and functional performance assessments were also conducted. Participants were randomly allocated in a single blind fashion to one of three groups: 1) Sedentary behavior fragmentation (SBF) (*n* = 13), 2) Single bout continuous light activity (LIPA) (*n* = 13), or 3) Control i.e. no lifestyle change (*n* = 8). All measures were taken at weeks 0 and 8.

### Dual X-ray absorptiometry scan

Participants arrived at the laboratory in a fasted state (10-h to 12-h overnight) and were taken into a private scanning room. Participants changed into a hospital style gown and had their height (to the nearest 0.01m) and body mass (to the nearest 0.1kg) measured using a stadiometer (Seca model 213 stadiometer, Seca, Germany), and digital scales (Seca model 873, Seca, Germany) respectively. A dual energy x-ray absorptiometry (DEXA) scanner (Hologic Discovery: Vertec Scientific Ltd, UK) was used to ascertain lean body mass. Briefly, participants were asked to lie in a supine position, avoiding any contact between the trunk and the appendicular mass [84] (whole body procedure, EF 8.4 lSv). The slow moving ‘arm’ of the DEXA scanner passed over the body over the course of 7 minutes. Hologic software was then used to draw segmental analysis lines through the skeleton along regions of interest (Arms, Legs, Total) [85, 86].

### Ultrasonography

**Figure 4:**
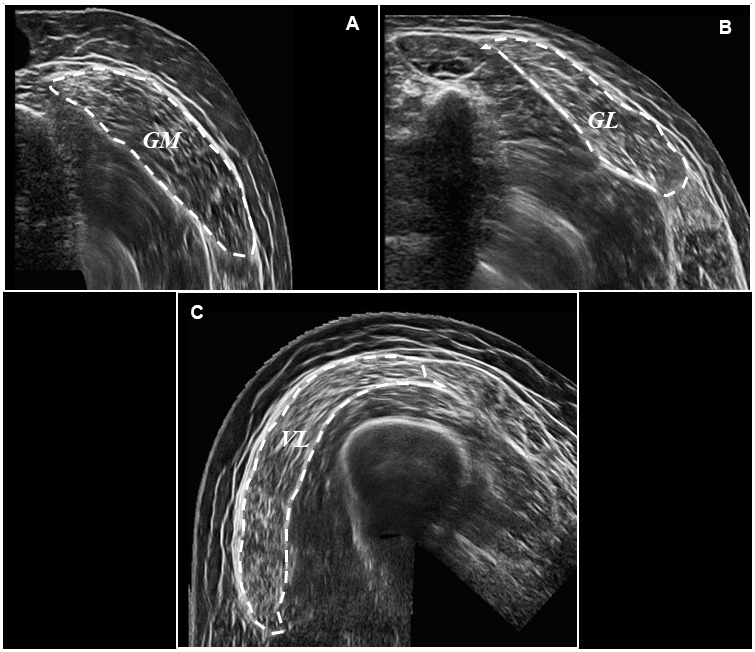
**Representative ultrasound images following panoramic ultrasound imaging.** Panel (**A**) represents a transverse image of *GM* CSA (outlined for effect) at 50% of muscle length, Panel (**B**) represents a transverse image of *GL* CSA (outlined for effect) at 50% of muscle length, and Panel (**C**) represents a transverse image of *VL* CSA (outlined for effect) also at 50% of muscle length.

Participants lay in a prone position, and rested for ~20 minutes to avoid fluid shifts [87, 88]. The ankle joint was then secured in neutral angle (0°) against a footplate. Participants were asked to remain still and relaxed, as Brightness-mode ultrasound (MyLab Twice, Esaote Biomedica, Genoa, Italy) was performed. Discrete muscle sites were marked by drawing a line from the medial to the lateral border of the *GM* and *GL*, at 25, 50, and 75% of each muscle’s respective length. Proximal and Distal endpoints of the *AT* were also marked, and length markers drawn in 1cm proximal increments from the calcaneal tuberosity. A novel panoramic imaging technique (panoramic view) granted an image of the *GM/GL* heads and thus anatomical CSA. Ultrasound panoramic imaging has previously been established as a reliable and valid method of CSA assessment when compared against magnetic resonance imaging [89, 90], and is sensitive to detect hypertrophic and atrophic alterations [83]. Briefly, the probe was moved with a constant speed and light pressure across the leg, to avoid compression during scanning. A Velcro strap was loosely attached (again to avoid compression) around the lower leg at each length marker to ensure the probe maintained the appropriate path and angle during each scan. Lastly, all ultrasound acquisition parameters were monitored, and consistency reproduced between scans. The ultrasound probe (7.5MHz linear array probe, 38 mm wide), was held perpendicular to the muscle. Once processing, the ultrasound probe was moved along the marked pathway, from the lateral to the medial border of the muscle (for representative images, please see Figure 4A, 4B) This procedure was repeated three times at each muscle site. The ultrasound probe was then positioned along the mid-sagittal line, at 50% of the GM muscle length, in order to record resting muscle architecture. Images of both resting fascicle pennation angle (FPA) and resting fascicle length (Lf), were then analysed using ImageJ (1.45s; National Institutes of Health). Three fascicles (defined from the deep to the superficial aponeurosis) of the GM were recorded and the mean value of both FPA and Lf determined. Linear extrapolation of fascicles was carried out where fascicles extended beyond the reach of the probe, as described previously [84]. This method has previously demonstrated good validity and reliability [33, 91].

*AT* length was measured as the distance from the distal gastrocnemius myotendinous junction to the calcaneal insertion. Subsequently, *AT* CSA was obtained from representative transverse images (Depth: 30mm; Frequency: 27Hz; Focal Points: 1) at 0, 1, 2 and 3cm, of *AT* length. Offline ultrasound analysis was performed using IMAGEJ (1.45 s; National Institutes of Health, Bethesda, MD, USA) in a non-blind fashion. Determination of tendon CSA using this method has previously demonstrated good validity and reliability [92, 93]. Participants then switched to a supine position, with the knee fully extended and the hip angle raised to 45°, on top of a 30cm platform. The proximal and distal insertions of the *VL* were identified and 50% of *VL* length marked on the skin. Three more panoramic images of the VL head and thus VL CSA were then obtained as described previously (for representative image, please see figure 4C).. *VL* muscle architecture (FPA and Lf) was then determined, as previously described for the *GM*.

### Ultrasound reliability

The same sonographer performed all scans and demonstrated excellent intra and inter day reliability. Specifically for the panoramic CSA imaging of the *GM*, *GL*, and *VL* the Intraclass Correlation Coefficient (ICC) was ~0.98, and the Coefficient of variation (CV%) ~4%, when reliability assessments were carried out on a subset of participants (*n*=8, 24% of total sample), comparing familiarization values to pre-test. Good inter day reliability was also observed for *VL* muscle architecture, specifically Lf (ICC = 0.96, CV% = 5%), and FPA (ICC = 0.87, CV% = 5%). For the *Achilles Tendon*, good inter day reliability was observed when CSA was examined at 0cm (ICC = 0.87, CV% = 7%), 1cm (ICC = 0.93, CV% = 6%), 2cm (ICC = 0.92, CV% = 6%), and 3cm (ICC = 0.76, CV% = 8%), and average of all sites (ICC = 0.97, CV% = 3%), when comparing tendon CSA at familiarization with pre-test values. Finally, good inter day reliability was also observed for GM muscle architecture, specifically FPA (ICC = 0.80, CV% = 4%), and Lf (ICC = 0.91, CV% = 67%), in a sub sample of participants (n=7, 21% of total sample).

### Calculation of muscle volume and physiological cross-sectional area

*GM* and *GL* muscles volumes were calculated by treating the muscles as a series of truncated cones [94, 95], through the construction of several CSAs taken at discrete muscle sites (25, 50 and 75% of *GM* and *GL* length). Each of the four truncated cones was calculated using the following equation:

$$muscle\;volume=\frac{1}{3}\cdot d\cdot [a+\sqrt{\left(a.b\right)}+b]$$

Where:

*d* is the distance between the two CSA’s (*a* and *b*)

The sum of the four cones provided *muscle volume* for *GM* and *GL*. *VL* muscle volume was calculated from a single CSA re-construction at 50% of *VL* length and extrapolated to calculate overall muscle volume. This method of calculating muscle volume from a single CSA has been validated previously [96]. Physiological cross-sectional area (PCSA) was then calculated for both *GM* and *VL* using the following equation, as described previously [84].

PCSA=muscle volume / Lf

### Postural balance assessment

The single balance postural test is well established in research using older persons, with documented reliability [55, 97]. Participants performed a single leg balance test with their eyes either open or with visual feedback removed through utilizing blacked out goggles to isolate proprioceptive feedback [72]. Each participant’s postural balance was tested on the leg they self-perceived to be their strongest. A number of measures were in place during assessment: (i) the researcher was present during all balance assessments; (ii) a soft chair was positioned behind the participants as a safety measure; (iii) participants hovered their hands above a height adjustable physiotherapy bed to begin the test before placing their hands by their side; (iv) in the event they felt they were going to lose their balance they would immediately place their hands back on the bed. This also marked the end of a particular trial along with raising the arms above head height or putting the non-balancing leg on the floor. Trial duration (up to a maximum of 30s) was recorded using a stopwatch. Three trials were performed with ~60s rest in-between. The average of the three trials for eyes open and eyes closed was then reported for each participant. Inter-day reliability was excellent for eyes open trials [Intraclass correlation co-efficient (ICC): 0.97%], and good for eyes closed trials (ICC: 0.75).

### Gait speed assessment

Gait speed was assessed through the timed “Up and Go” test (TUG) [98, 99]. In an attempt to reduce TUG variability [100], the height of an adjustable stool was standardized to the length of each participants lower leg (distance in cm from the tibio-femoral junction, to the bottom of the footwear). The time taken between rising from and returning to the seated position was accurately monitored with a modified pressure sensor (Tekescan, South Boston, USA), and corresponding software. The sensor was attached to the surface of the chair in a manner that allowed accurate timing (0.01s) but did not impede the participants comfort whilst seated. Once instructed, participants rose from the chair, and walked at a maximum self-selected pace up to a box marked out on the floor with masking tape (approximately 6m away), before returning to the seated position. Total time was divided by the total course distance (12m) in order to calculate average gait speed [metres per second (m/s)]. The test was repeated 3 times with 60s rest in-between, and the average of three trials reported. Gait speed assessment exhibited excellent inter-day reliability (ICC: 0.91).

### Comprehensive sarcopenia definition

DEXA derived appendicular lean body mass was divided by body height to provide a relative indicator of muscle quantity, termed sarcopenic index. Previously determined cut off points for both sarcopenic index and gait speed [74], were then used to classify participants into one of four categories, 1. Non-sarcopenic (sarcopenic index ≥5.5kg/m^2^ and gait speed >0.8 m/s), 2. Pre-sarcopenic (sarcopenic index <5.5kg/m^2^ and gait speed >0.8 m/s), 3. Low functional performance (sarcopenic index ≥5.5kg/m^2^ and gait speed ≤0.8 m/s), and 4. Sarcopenic (sarcopenic index <5.5kg/m^2^ and gait speed ≤0.8 m/s).

### Physical behavior interventions

The purpose of the two intervention groups was to manipulate the method for displacing sedentary behavior time with added daily LIPA (45-50 mins). Both intervention groups were provided with a booklet, which contained simple LIPA suggestions compiled from the compendium of physical activities [101]. Participants were explicitly told to continue performing any pre-existing MVPA routines (e.g., exercise classes, etc). Throughout the 8-week intervention period all participants received fortnightly home visits from a member of the research team, to check on the progress of the intervention. Participants daily sedentary behavior, LIPA, and MVPA were assessed at baseline and the final intervention week, with a thigh mounted GENEActiv original triaxial accelerometer (GENEA, Activinsights Ltd, Kimbolton, UK), and a previously validated algorithm [102]. Participants were classified as sedentary if average daily sedentary time was ≥8h/day, as sedentary time appears to be exponentially hazardous above this threshold [2, 3].

*SBF group*: Participants were told that the purpose of their intervention was to reduce the amount of time spent performing sedentary behavior (sitting, lying, or reclining) especially in prolonged uninterrupted bouts. Participants were instructed not to perform sedentary behavior for more than 30 minutes at a time, and that for every 30 minutes of sedentary behavior performed the participant should stand up and perform 2 minutes of upright LIPA (general ambulatory walking, side to side shuffling, washing dishes etc).

*LIPA group:* Participants were informed that the purpose of their intervention was to increase the amount of time spent performing LIPA whilst maintaining habitual routines. Participants were instructed to perform a continuous single bout of 45-50 minutes LIPA (general ambulatory walking, side to side shuffling, washing dishes etc), every day for the duration of the 8-week intervention.

*Control group*: Participants who were randomly allocated to the control group were specifically instructed to maintain their habitual routine. Control participants were told that the overall purpose of the study was to study the link between health and habitual activity profiles.

### Statistical analyses

Statistical analyses were carried out using SPSS (Version 25, SPSS Inc., Chicago, IL, USA). Parametricity was checked through the Shapiro–Wilk test to determine data normal distribution and the Levene’s test to determine equality of variances between groups. If parametric assumptions were met, baseline group differences were examined by a one-factor analysis of variance (ANOVA) (SBF, LIPA, CON) with post-hoc pairwise comparisons conducted using the Least Significant Difference. The effects of the interventions were determined using 2×3 split plot ANOVA (2 phases and 3 groups) or 2×4×3 (2 phases, 4 anatomical sites and 3 groups) split plot ANOVA depending on the outcome variable. Furthermore, linear regression analysis was performed on the relative changes from baseline for each muscle-tendon complex/ lean body mass outcome, and the relative changes from baseline for each functional performance outcome. GM muscle architecture data was not collected for 2 participants, meaning such analyses were carried out on a sub-sample (n=32). In cases of heteroscedasticity in variances, the Greenhouse Geisser correction was applied. In cases of non-normal distribution within group comparisons were made using the Wilcoxon-Sign Rank test, whilst, between group differences utilized a Kruskal-Wallis non-parametric equivalent of ANOVA (SBF, LIPA, CON) with post-hoc pairwise comparisons examined by Mann-Whitney U test. Chi-squared analysis was used to investigate nominal variables. Data are reported as Mean±SD (or Median, IQR for non-parametric data). Statistical significance was accepted when *P*<0.05. Furthermore, a statistical trend was deemed to be present when *P* was in the range of between 0.05 to 0.10. Study power (β) and effect size (ɳ_p_^2^) are also reported where P is significant.
